# Effect of cold and frozen temperatures on artisanal goat cheese containing probiotic lactic acid bacteria isolates (*Lactobacillus plantarum* TW14 and *Lactobacillus rhamnosus* TW2)

**DOI:** 10.14202/vetworld.2019.409-417

**Published:** 2019-03-16

**Authors:** Triana Setyawardani, Juni Sumarmono, Kusuma Widayaka

**Affiliations:** Department of Animal Production, Faculty of Animal Science, Jenderal Soedirman University, Purwokerto, Indonesia

**Keywords:** cheese, lipolysis, physicochemical, proteolysis

## Abstract

**Aim:**

The research was conducted to determine the effect of temperature and storage duration on the physicochemical, lipolytic, microbiological, and proteolytic characteristics of goat cheese made using *Lactobacillus plantarum* TW14 and *Lactobacillus rhamnosus* TW2 bacteria.

**Materials and Methods:**

The cheese was stored at 4°C and −20°C for 0, 15, 30, 45, and 60 days. Observations were made on its physicochemical, lipolysis, and microbiological characteristics. The proteolysis pattern was measured with sodium dodecyl sulfate-polyacrylamide gel electrophoresis.

**Results:**

The protein, fat, ash and total solids levels of cold-stored cheese were higher than the frozen-stored one. The frozen-stored cheese’s free fatty acids (FFA) and acid degree value (ADV) levels are lower than those of the cold-stored cheese as indicated by the partial lipolysis event. The total yeast in the frozen-stored cheese is lower than that in the frozen-stored cheese. Finally, the electrophoresis profile indicates that proteolysis of the frozen-stored cheese is formed since there have been detected α_s1_-casein, α_s2_-casein, β-casein, and κ-casein in the casein breakdown during the 60-day storage.

**Conclusion:**

The physicochemical characteristics of cold-stored cheese are better than the cheese stored at frozen temperature. However, frozen-stored cheese produces lower FFA and ADV than cold-stored cheese and lipolysis occurs only partially.

## Introduction

Goat cheese has been commonly produced in many countries. Many use probiotic lactic acid bacteria (LAB) of *Lactobacillus* spp. and *Bifidobacterium* spp., which have been proven capable of improving health. Previously, we isolated and identified two indigenous LAB (*Lactobacillus plantarum* TW14 and *Lactobacillus rhamnosus* TW2) from the milk of local Indonesia goat breeds [1], which were proven resistant to low pH and bile salt 0.3% and capable of colonizing in the intestinal epithelium, possessing antimicrobial property [2]. Both types of LAB are used as a starter to produce cheese which is expected to generate specific characteristics in goat cheese different from any existing goat cheese. *L. plantarum* TW14 and *L. rhamnosus* TW2 are used in this work to manufacture cheese from goat milk. The use of mixed probiotic bacteria improves the viability of probiotic in cheese when compared to a single strain, [3], which were similar to the observation on whey cheese [4].

Cheese is potential for delivering probiotic LAB to the gut; however, storage temperature and duration during distribution and display might affect the survivability of the probiotics and cheese characteristics. Previous study [5] was only limited on storage under room and cold temperature of the cheese. This study was performed to endeavor the effect of frozen storage condition. That storage condition can maintain food quality for a longer time. The advantage is that the food could be distributed in a longer distance than other methods. In addition, the room temperature storage accelerates the ripening of ripened cheese type [5]. Under cold and frozen storage, cheese texture becomes finer, and the consistency solidifies, the flavor is formed more perfect, and the LAB metabolite is produced. These changes occur due to the proteolysis process and interaction of LAB in the cheese matrix [6-8]. The LAB viability during storage is also influenced by its resistance to the storage temperature. In frozen temperature, ice crystals are formed and will disrupt the LAB growth as well as decrease the viability. The previous study proved that the stored cheese on cold temperature also changed its metabolite and LAB viability [3]. The viability could be maintained up to 9.69±0.12 log colony-forming unit (CFU)/g. The storage duration contributed to the amount of volatile compounds produced and the biochemical changes [5]. This biochemical process and microbial interaction during the storage will be beneficial since it will improve the flavor and texture of cheese; both are important in improving the probiotic cheese quality. The existence of probiotic bacteria is expected to improve the functional property of the cheese as well since the consumption of probiotic in an adequate amount will improve the body’s physiological function.

The novelty of this study is that we used indigenous LAB isolated from the milk of local Indonesia dairy goats. The isolates (*L. plantarum* TW14 and *L. rhamnosus* TW2) were particularly promising as probiotics. Incorporation of these LABs into cheese would create an opportunity to produce a kind of probiotic cheese, which has functional properties. Therefore, this study aimed to investigate the effects of storage condition (cold and frozen) as well as storage duration on the physicochemical, lipolytic, microbiological, and proteolytic characteristics of goat cheese manufactured using *L. plantarum* TW14 and *L. rhamnosus* TW2 bacteria.

## Materials and Methods

### Ethical approval

Ethical approval was not needed for this study.

### Materials

The probiotic culture of *L. rhamnosus* TW2 and *L. plantarum* TW14 was isolated previously [1]. Commercial animal rennet, microbiology media of de Man Rogosa and Sharpe Broth (MRSB) (Difco Laboratories Detroit, MN, USA), phosphate buffer saline (Sigma Aldrich, Missouri, USA), and chemicals were collected for laboratory analysis. Tools used in this experiment consisted of cheese manufacturing tools, an incubator, proximate analysis apparatus, and sodium dodecyl sulfate-polyacrylamide gel electrophoresis.

### Methods

#### Culture starter making

The *L. plantarum* TW 14 and *L. rhamnosus* TW2 isolates were isolated from goat milk [1]. Each of these isolates was taken one ose, and then, it was grown in MRSB for 24 h at 37°C until it was turbid, which means that isolate concentration is about 10^8^ CFU/mL and ready to use as a starter.

#### Cheese production process

Fresh goat milk was obtained from a farmer in Ajibarang (2 h from the laboratory), then immediately pasteurized at 62°C for 30 min, and then cooled down to 40°C. The *L. plantarum* TW14 and *L. rhamnosus* TW2 isolates (10^8^ CFU/mL each) were added at 5% concentration of total milk (v/v) with 1:1 ratio for each isolate, then incubated it for approximately 4 h until its pH decreases to about 6.1, then added the rennet at 0.06 µl/L milk, and let it settle until a pudding-like lump is formed. The lump was cut and heat at 40°C for 10 min and then separates the solids and fluids by filtering. The solids were pressed and stored at cold (4°C) and frozen (−20°C) for 60 days.

#### Chemical composition

The protein (Kjeldahl’s method), fat (Babcock’s method), moisture for dry matter, and ash were measured according to the standards of AOAC [6].

### Salt level

The salt level of the cheese was determined using titration with AgNO_3_ [7].

### pH

The pH was measured using a pH meter (Hanna Instrument 8519, Incofar, Modena, Italy), and the cheese amounting to 10 g was blended into 10 mL distilled water [8].

### Acid degree value (ADV)

ADV was determined as follows [9], approximately 10 g of sample was ground, homogenized, and placed into a Babcock cheese bottle for fat extraction. 1 mL of the final extracted fat was titrated against the standard alcoholic 0.02N KOH (Merck, Darmstadt, Germany) solution. Calculation of ADV was performed using the following formulation:


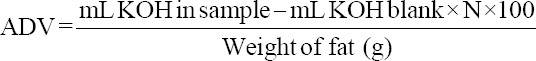


where N = normality of KOH solution in methanol (Merck, Darmstadt, Germany)

### Free fatty acids (FFA)

The method to quantify FFA was based on titration of NaOH (Merck, Darmstadt, Germany) that was described by Park [10]. The cheese was ground, and then, 10 g of them were added with 50 mL of 96% alcohol (Merck, Darmstadt, Germany) and 2 mL of phenolphthalein indicator (Merck, Darmstadt, Germany). The mixture was then titrated with 0.1N NaOH until pink color appeared and made sure that it was stable for 30 s. The calculation was carried to the formula below:

FFA = mL NaOH titrated for a sample – mL NaOH titrated for the blank sample × N × 100

Weight of sample (g)

where N = normality of NaOH solution

### Quantification of cheese LAB and yeast

About 20 g of sample was mixed with 180 mL of 2% sterile sodium citrate solution (Sigma Aldrich, Missouri, USA) (w/v) and homogenized in Stomacher (Fisher Scientific, Hampton, NH) at 12,000 rpm for 3 min. 1 mL of the homogenized solution was taken and serially diluted up to 1:10^8^. The sample of the three highest dilutions specifically 10^6^, 10^7^, and 10^8^ CFU/mL was taken 1 mL each aseptically and poured into a sterile Petri dish and MRSA (Difco Laboratories Detroit, MN, USA). Media was added and then incubated at 37°C for 48 h to calculate the amount of LAB by total plate count method, and the total yeast is grown in PDA (Difco Laboratories Detroit, MN, USA) followed by same quantification method [8].

### Proteolysis profile

Proteolysis profile was performed using gel electrophoresis as described by Laemmli [11] with some modifications. 1 g of cheese was defatted by extracting with diethyl ether, dissolved in solution containing 1 mL of EDTA (Sigma Aldrich, Missouri, USA) (1%), 1 mL of sodium deoxycholic (Merck, Darmstadt, Germany) (1%), and 5 mL of urea (Sigma Aldrich, Missouri, USA) (50% w/v). The pH of the solution was set at 7.0 and ripened at 4°C for 24 h. The sample was then diluted with sample buffer (Sigma Aldrich, Missouri, USA) 1:4 v/v and denaturated in 95°C for 5 min. The samples were loaded to 12% gel (acrylamide: bis-acrylamide; 29:1) (Merck, Darmstadt, Germany) along with Chromatein Protein Ladder (Vivantis Technologies, Selangor, Malaysia); then, the electrophoresis was performed in 150 V for 30 min. These processes were followed by coomassie staining (Sigma Aldrich, Missouri, USA), and documentation.

### Statistical analysis

The data were collected and presented as means±standard deviation. The procedure of general linear model was employed, followed by Duncan’s multiple range test for mean comparison.

## Results and Discussion

**Figure-1:**
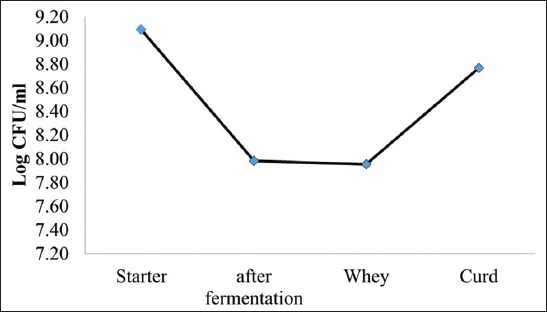
Total count of lactic acid bacteria in each manufacturing step.

**Figure-2:**
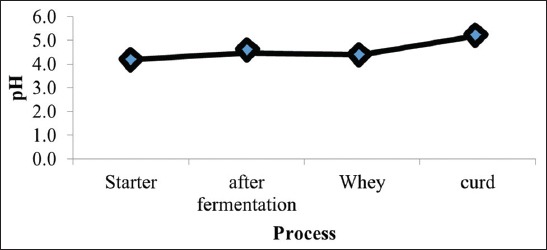
pH values in each manufacturing step.

The milk used as the raw material for cheese production has a total number of bacteria 3.97 log CFU/mL, and on pasteurization, it reduces to 2.81 log CFU/mL. The amount of probiotic LAB used collectively, i.e., *L. plantarum* TW14 and *L. rhamnosus* TW2 in the culture starter is shown in Figure-1. In the cheese curd, LAB amounting 8.77 is obtained before the cheese is given different temperature and storage duration treatments. The fresh milk used has pH 6.8 and the starter has pH 4.2 during the incubation process. The milk pH decreases to 4.5 after the starter is added. During the cheese production process, the whey and curd are separated with the whey pH 4.4 and the curd pH 5.2 (Figure-2).

### 

#### Cheese chemical composition

The goat cheese was produced using *L. plantarum* TW14 and *L. rhamnosus* TW2 probiotic isolates as its starter. These isolates were isolated from the milk of goat of Ettawa crossbreed, and its chemical composition is shown in Table-1.

**Table-1:** Chemical characteristics of goat milk cheese at different temperatures and storage durations.

Temperature (°C)	Storage (days)	Protein (%)	Fat (%)	Water (%)	Ash (%)	Dry matter (%)	Salt (%)	pH
Frozen (−20)	0	14.25±1.53^ab^	23.60±0.77^c^	55.95±1.93^ab^	1.75±0.08^a^	44.05±1.93^bc^	1.130±0.35^a^	5.04±0.07^b^
	15	14.84±1.16^a^	20.08±2.12^ab^	56.35±3.56^bc^	3.72±0.50^c^	43.65±3.56^ab^	1.316±0.46^b^	4.56±0.25^a^
	30	15.84±1.75^ab^	23.51±3.09^bc^	53.096±4.01^a^	2.68±0.38^b^	46.90±4.01^bc^	1.483±0.39^ab^	4.53±0.17^a^
	45	14.88±0.93^a^	21.27±0.96^a^	57.33±0.62^c^	2.37±0.01^c^	42.67±0.62^a^	2.473±0.63^b^	4.29±0.23^a^
	60	17.38±1.08^b^	23.18±2.72^abc^	49.80±3.18^a^	3.02±0.45^b^	50.19±3.18^bc^	2.236±0.82^b^	5.04±0.25^b^
Cold (4)	0	21.02±1.25^ab^	29.36±1.69^c^	40.19±3.17^ab^	3.00±0.38^a^	59.80±3.17^bc^	1.840±1.03^a^	4.78±0.16^b^
	15	17.57±1.70^a^	27.21±0.63^ab^	46.43±3.41^bc^	4.12±0.41^c^	53.57±3.41^ab^	3.183±0.36^b^	4.34±0.18^a^
	30	19.87±0.90^ab^	28.08±1.40^bc^	40.46±2.30^a^	3.96±0.32^b^	59.53±2.30^bc^	2.386±0.68^ab^	4.42±0.23^a^
	45	18.42±3.06^a^	23.92±3.36^a^	48.28±5.51^c^	6.10±0.71^c^	51.71±5.51^a^	3.373±0.39^b^	4.51±0.04^a^
	60	21.32±1.06^b^	26.02±2.46^abc^	42.70±3.51^a^	3.70±0.19^b^	57.29±3.51^bc^	3.180±1.84^b^	5.29±0.22^c^

Results are expressed as mean±standard deviation (n=3). Mean without common superscript (a-c) in column is significantly different (p<0.05)

Any cheese stored in cold temperature will have protein contents, and the cheeses made using *L. plantarum* TW14 and *L. rhamnosus* TW2 starter culture have protein contents from 17.57% to 21.32%. The storage duration of cheese at different temperatures has a significant influence (p<0.05), and the different storage durations have a significant influence (p<0.05) on the cheese’s protein content.

The fat contents as found by this research range from 20.08±2.12% to 29.36±1.69%, with the lowest fat content found in the cheese treated with 15-day frozen storage and the highest averaged in the cheese treated with no storage at cold storage. The cheese storage temperature, the storage duration up to 60 days, and the interaction between temperature and storage duration have an insignificant influence (p>0.05) on the cheese’s fat content. The cheese with no storage duration produces the highest fat content, and it decreases during the storage.

The ash content as found in the research ranges between 1.75.±0.08% and 6.10±.0.71%, with the highest ash content value found in the refrigerator temperature treatment for 45-day storage, i.e., at 6.10±.0.71%. The lowest ash content is found in the cheese before its storage in freezer storage, i.e., 1.75.±0.08%. The cheese storage temperature, the storage duration up to 60 days, and their interaction have a significant influence (p<0.05) on the cheese ash content. The produced ash content during the 30-day storage has the highest at 3.09%. The obtained value is lower than that found by Laemmli [11], i.e., 4.37%.

The water content of the cheese stored in freezer is higher than that stored at refrigerator temperature, with the highest found in the cheese stored for 45 days in both cheeses, frozen and cold storage, i.e., respectively, at 57.33 and 48.28, and the same applies to the total solid cheese as well. Temperature and storage duration influence the cheese salt content (p<0.05), where the salt content is higher in the cheese stored in the refrigerator than that of the cheese stored in the freezer. The longer the duration, the higher the salt content that the cheese will have.

The cheese with *L. plantarum* TW14 and *L. rhamnosus* TW2 obtains water content ranging from 40.19±3.17 to 57.33±0.62%, and it can be classified as soft cheese. The cheese’s total solids range from 42.67±0.62 to 59.80±3.17%. The lowest total solids are found in the cheese stored at freezer temperature for 45 days. The result of variance analysis shows that cheese storage temperature and storage duration up to 60 days have a significant influence (p<0.05). An increase in the total solids during the cheese ripening is also found by the research conducted by Dervisoglu [12], which is followed by a significant decrease in the cheese water content. The pH of cheese produced using mixed culture of *L. plantaru*m TW14 and *L. rhamnosu*s TW2 was between 4.29±0.24 and 5.29±0.22 after 30-day of storage. The probiotic LAB activity during the processing and product storage can be observed by seeing the change in pH and LAB amount. The result of analysis of variance (ANOVA) indicates that cheese storage temperature and storage duration up to 60 days have a significant influence (p<0.05) on the cheese pH value. The cheese pH value is influenced by storage time. The longer the cheese stored at freezer and refrigerator temperatures, the lower the pH value. However, the pH value tended to increase after 60-day of storage.

The protein and total solid contents of cheese samples stored under refrigeration temperature is higher than those of the stored under frozen condition. The increased in total solids of cold-stored cheese are influenced by the components which make up the total solids and the decreased water when the cheese is stored. This result confirms the study by Mushtaq *et al*. [13]. The increased total solids during the cheese ripening period are due to water evaporation during the cold storage. This confirms the previous study, namely on Kulek cheese. The decreased water content during the cheese cold storage occurs due to the cheese’s syneresis process. The decreased water content when the cheese is stored [12], generally because the salt added to the cheese production process will increase the cheese’s total solids, and hence, the water content decreases.

During the cheese storage, LAB keeps on growing, develops with the cheese substrates, and produces metabolites in the form of lactic acid which is capable of decreasing the cheese’s pH. The low pH value of the cheese during storage plays a role in helping to create a non-conducive environment for other bacteria to grow and develop, particularly the pathogen and deteriorating bacteria. At the end of the product storage, there is no increase in the pH value. This indicates that the LAB cultures used are still actively performing metabolism activities, resulting in active production of lactic acid which, in turn, accumulates the lactic acid and the cheese’s pH keeps decreasing. In general, increment of pH is contributed to the activities of mold and yeast in using lactic acid as the source of carbons, and to the proteolysis process which releases a large amount of alkaline components. These results confirm that cheeses stored for 60 days have an increased pH value.

#### Cheese lipolysis

The FFA value shows an increased lipolysis activity, and thus, the concentration of FFA content also increases. The acid number is tightly related to FFA contents. ADV is an estimated number of FFA existing in fat sample calculated as the hydrolysis lipolysis index in milk products.

The storage temperature influences the amount of the cheese FFA and ADV values, where the cheese stored in the refrigerator has higher FFA and ADV values those stored at frozen temperature. In the frozen storage, the FFA and ADV values decrease in the 60-day storage duration, and in the cheese stored in refrigerator, the FFA and ADV values increase after 45-day storage. This experiment showed that the FFA content of the cheese after 30-day of storage was 3.09±1.37 and increased to 11.95±0.33 after 60-day of storage at refrigerator temperature. During frozen storage, the FFA content did not increase; it even decreased when the cheese is stored until the 30^th^ day. The result of ANOVA shows that the cheese storage, duration, and their interaction have a significant influence (p<0.05) on the cheese FFA value. During the frozen storage (−20°C), the FFA value decreases significantly, and the 60-day storage has the lowest FFA value, i.e., 2.05.

This differs from the FFA value of cheese stored in the refrigerator (4°C), where the cheese FFA value increases after 30-day storage, i.e., in the 45^th^ and 60^th^ days. For 60 days, the highest FFA value is 11.95, indicating that lipolysis occurs in the cheese stored at cold/refrigerator temperature as shown by the magnitude of FFA value at the end of the cheese storage, even though this FFA value in 60-day storage is not different from the cheese before it is stored, i.e., 7.21. The FFA value of cheese stored at cold temperature in 0-, 15-, 30-, 45-, and 60-day storages is 7.21, 10.28, 3.09, 11.13, and 0.42±0.02, respectively. In the 30-day storage, the cheese FFA value decreases, which is enabled by the decreased activity of lipoprotein lipase as compared to the 45- and 60-day storages.

The research result shows that the acid number of the cheese after being stored for 60 days ranges between 0.11±0.05 and 11.95±0.33. During the storage, the FFA content does not increase; it even decreases when the cheese is stored until the 30^th^ day. The result of variance analysis shows that cheese storage temperature, storage duration up to 60 days, and interaction have a significant influence (p<0.05) on the cheese’s acid number. The ADV value in the cheese stored at frozen storage decreases at the end of the storage, i.e., at 0.07, yet in the cold-stored cheese, the cheese’s ADV value increases in the 60-day storage, i.e., at 0.42. The ADV result has the same tendency as the cheese FFA value. In frozen storage, the ADV is very low, indicating that very small lipolysis occurs as compared to the cold temperature storage.

At the end of the frozen storage, the FFA value does not increase. This indicates that there is no damage to the fat in the cheese stored for 60 days. During the freezing time, no fat globule in the cheese is damaged due to the formation of ice crystal when the cheese is kept in frozen storage. The milk fat naturally exists in the fat globule which is reinforced by the phospholipid and membrane-rich of proteins undamaged by the formation of crystal during the freezing. During the freezing, the FFA value does not increase. The cheese’s FFA value in cold storage is higher than that in frozen storage, and at the end of cold storage, the highest FFA value is 7.21. The FFA value shows that lipolysis occurs in the cold-stored cheese. The lipolysis in the cheese during the storage is influenced by some factors such as lipase enzyme, factors existing within the milk, rennet, and the microbes developing during the cheese ripening process [14].

Some researchers report that the lipolysis process in milk and its products can occur in different ways, namely: (1) induced lipolysis, (2) spontaneous lipolysis, and (3) lipolysis caused by microbes. The induced lipolysis is influenced by several factors including the stirring, separation, mixing, presence of air, homogenization, and changes in temperature such as freezing, thawing, storage, and processing [15]. Spontaneous lipolysis occurs with two main factors, namely processing and the factors from the livestock themselves. Lipolysis by microbe is caused by several bacteria contaminating the product. The microbe produces lipase which generates rancid flavor. The decreased total fat measured, particularly triacylglycerol, constitutes the cause of lipolysis by lipoprotein lipase. The full fat cheeses stored under cold temperature have higher FFA value than low-fat cheese. This is due to the lipolytic enzyme activity in lipoprotein deriving from milk indigenous enzyme or microbial enzyme which may damage the milk fat globule membrane when the milk is separated from the fat, during the processing and storage [9].

The cheese’s ADV value has the same tendency as the FFA value, wherein cold storage, the ADV increases at the end of storage, i.e. at 0.42. This result is far below the ADV in feta cheese which is ripened for 60 days at 3-4°C, i.e. at 1.04 [15]. The lipolysis is indicated by the increase in the cheese sample’s ADV value (Table-2). The table indicates that the low ADV value in both cheese storages for 60 days shows the low lipolysis process. The ADV value produced in this research is lower than that what is found by Oliveira [16] and confirms the result obtained by Nouira *et al*. [9]. In general, the low ADV value shows the less lipolysis occurring in the cheese which is in line with the FFA results.

**Table-2:** FFA and ADV contents of goat milk cheese stored at different temperatures and storage durations.

Temperature (°C)	Storage (days)	FFA (%)	ADV (%)
Frozen (−20)	0	7.62±1.42^a^	0.27±0.05^a^
	15	7.66±0.51^a^	0.27±0.01^a^
	30	4.35±0.39^b^	0.15±0.01^b^
	45	6.73±0.60^a^	0.23±0.02^a^
	60	2.05±0.55^b^	0.07±0.01^b^
Cold (4)	0	7.21±0.91^a^	0.25±0.03^a^
	15	10.28±0.75^a^	0.36±0.02^a^
	30	3.09±1.37^b^	0.11±0.04^b^
	45	11.13±4.21^a^	0.39±0.14^a^
	60	11.95±0.33^a^	0.42±0.02^a^

Results are expressed as mean±standard deviation (n=3). Mean without common superscript (a-c) in column are significantly different (p<0.05). FFA=Free fatty acid, ADV=Acids degree value

The main factors influencing cheese lipolysis are fatty acid composition, lipolytic enzyme, lipolytic microbe, water, temperature, storage duration, oxygen, and surface area. The lipolysis in goat cheese can be estimated from ADV and the cheese FFA concentration.

#### Cheese’s microbiology

The research result showed that the amount of the cheese’s LAB after being stored for 60 days ranges between 8.27±0.30 log CFU/g and 9.143±0.50 log CFU/g (Table-3). During the storage, the amount of total average LAB is 8.81 log CFU/g, meaning that no increase is found. It even decreases when the cheese is stored until the 30^th^ day. Cheese storage temperature, the storage duration up to 60 days, and the interaction have an insignificant influence (p>0.05) on the cheese’s LAB.

**Table-3:** Microbiological characteristics of goat milk cheese stored at different temperatures and storage durations (log CFU/g).

Temperature (°C)	Storage (days)	Amount of total bacteria	LAB	Yeast
Frozen (−20)	0	8.173±1.73	9.027±0.23^a^	4.757±0.13^b^
	15	8.510±0.11	8.607±0.09^a^	3.027±0.39^ab^
	30	8.613±0.70	8.427±0.30^abc^	2.393±0.56^a^
	45	8.540±0.36	8.580±0.29^abc^	3.673±0.50^b^
	60	8.417±0.25	9.006±0.35^bc^	5.367±0.19^c^
Cold (4)	0	9.297±0.66	8.813±0.08^a^	4.123±0.48^b^
	15	8.733±0.12	8.617±0.13^a^	5.570±0.01^ab^
	30	8.777±0.04	8.847±0.15^abc^	5.553±0.07^a^
	45	8.467±0.04	9.143±0.50^abc^	5.430±0.21^b^
	60	5.060±0.08	9.093±0.14^bc^	5.457±0.04^bc^

Results are expressed as mean±standard deviation (n=3). Mean without common superscript (a-c) in column is different (p<0.05). LAB=Lactic acid bacteria, CFU=Colony-forming unit

The frozen storage in goat cheese is highly desired to maintain the cheese quality during distribution and marketing. Information on goat cheese’s microbiological quality during its storage in freezer and at cold temperature is still limited, particularly to discover the microbiological characteristics as seen from the total bacteria calculated in the cheese stored in freezer and refrigerator where both have the same tendency, yet their storage durations influence the total bacteria (p<0.05). The total bacteria of goat cheese which is on 0-day frozen storage is lower than on cold temperature storage which indicates that bacteria is more resistant to cold temperature than to frozen temperature before the cheese is stored. At cold temperature, bacteria can still grow even though its progress is slow. In the 60-day storage, the number of bacteria decreases up to 3 log cycle. This indicates that the total bacteria calculated in the cheese after 45-day storage begins to be dominated by LAB, and hence, the number of bacteria decreases significantly. This result is supported by the data on the amount of LAB experiencing an increase after 30 days of cold storage duration, i.e., at 9.143 log CFU/g. The bacteria existing in the cheese are secondary microflora deriving from milk or another contaminant during the cheese production process. The total LAB of the cheese stored at both freezer and refrigerator temperatures is relatively the same. However, in the 60-day storage duration, the total LAB increases significantly (p<0.05) at both storage temperatures. The increased LAB in the cheese stored at cold temperature begins since the 30^th^ day of storage, and in the frozen-stored cheese, the total LAB increase begins after the 45^th^ day.

The total yeast in the cheese is 2.393±0.56-5.570±0.0 with a total average of 4.532±1.15 log CFU/g. The storage temperature has a significant influence (p<0.05) on the cheese’s total yeast. The total yeast in frozen storage is lower than that in refrigerator storage, yet it has the same tendency as in the cheese store for 60 days which has the highest total yeast value. The total yeast decreases on the 15^th^ and 30^th^ days of the storage duration, yet it re-increases in the 45^th^ day. In the frozen storage, the fact that ice crystal is formed has caused the bacteria, including the yeast, to take more time to adapt to the new environment, resulting in its decreased number. In the 45^th^ day of the storage, it begins to be able to adapt, and the total yeast increases until the end of the 60-day storage. This phenomenon is a little bit different from the cheese stored at cold temperature. Before its storage, the cheese has the least total yeast, i.e., 4.123 log CFU/g and it increases during the 60-day storage. In the cold storage, the yeast can still grow and multiply in the cheese matrix, where the yeast in the cheese constitutes a contaminant which possibly comes from the milk or during processing.

For the 60-day cheese storage at cold temperature, the total bacteria decreases drastically, i.e., 5.06 log CFU/g as compared to the cheese stored at frozen. At cold temperature, the bacteria can still slowly grow. On the 60^th^ day of storage, the number of bacteria decreases up to 3 log cycles. This indicates that the total bacteria calculated in the cheese after 45-day storage begins to be dominated by LAB, and hence, the number of bacteria decreases significantly. This result is supported by the data on the amount of LAB experiencing an increase after 30 days of cold storage duration, i.e., at 9.14 log CFU/g. The bacteria existing in the cheese are secondary microflora deriving from milk or another contaminant during the cheese production process.

This shows that, in cold temperature storage, LAB still performs metabolism and can multiply and produces higher viability than in the frozen-stored cheese. In addition, cheese is one of the ideal probiotic bacteria/LAB carriers as compared to other fermentation products because cheese has pH close to buffer pH. In addition, the LAB in cheese is protected within the cheese matrix which contains fat, protein, and others. The cheese’s water content is relatively lower than other fermentation products such as yogurt. LAB are the ones dominating when the cheese is stored with the average total LAB being 8.81 log CFU/g. Meanwhile, yeast also grows during the cheese storage at a total average of 4.53±1.15 log CFU/g. In this research, the yeast grows more slowly than LAB.

The existence of yeast in cheese product is possible due to the contamination during the cheese production process, that is, in the curdling process, during the cheese salting in salt solution and the tools used. The number of yeast calculated in the cold-stored cheese is higher than that in frozen-stored cheese. This result confirms the research conducted by Park [17] which finds that frozen-stored cheese has a fewer number of yeast than before it is stored. The existence of yeast in the cheese is possible due to cross-contamination during processing and handling. The increased number of yeast at the end of storage duration at both temperatures is because the yeast can metabolize the lactic acid produced by the LAB, and thus, the cheese’s pH at the end of storage increases. The yeast growth is possible due to contamination during the curd formation process, during salting and tool contamination. During the storage, the number of yeast increases significantly. This result confirms the research conducted by Kołakowski [18]. During ripening, the microflora population increases until 4-week storage and slowly decreases until 12 weeks. The total LAB increases at 0.5 log cycle, and at the same time, the yeast also increases from 4 log to 6 log/g after 12 weeks of ripening period [18]. Ong *et al*. [19] indicate that the number of *Lactobacillus acidophilus* and *Lactobacillus casei* is 8 log CFU g^−1^ in cheddar cheese for 6-month storage period. Souza *et al*. [20] tested the stability of *L. acidophilu*s in Brazilian fresh cheese (Minas fresh cheese) for 3 weeks [21]., and proved the stability of *L. acidophilu*s and *L. case*i mixture in soft cheese stored for 8 weeks which can maintain its number (9 log CFU g^−1^). The viability of *Lactobacillus paracasei* A13 increases in the cheese stored at 5°C [22].

#### Cheese change during the storage in electrophoresis

Proteolysis plays a role during the cheese ripening process by producing aroma through the formation of peptide and amino acid and the cheese texture change from protein matrix breakdown [23].

The cheese stored at frozen temperature has a molecular weight between 10 and 260 kDa, where the casein group of the cheese falls within the range of BM 17-28 kDa. As long as the cheese is stored at frozen temperature for 60 days, the band belonging to the BM 17-28 range remains. The group belongs to α_s1_-casein with BM 23 kDa, α_s2_-casein with BM 25 kDa, β-casein with BM 24 kDa, and κ-casein with BM 19 kDa [24]. The profile seems to be overlapping as no perfect separation from the band has occurred yet, particularly within the BM 17-28 kDa range. All bands were still detected in all samples until the end of 60-day storage. This shows that the breakdown by plasmin occurs only partially, and hence, no protein hydrolysis occurs in the samples. This partial hydrolysis in the cheese samples is possible due to the low pH as in the case of salted cheese [21].

**Figure-3:**
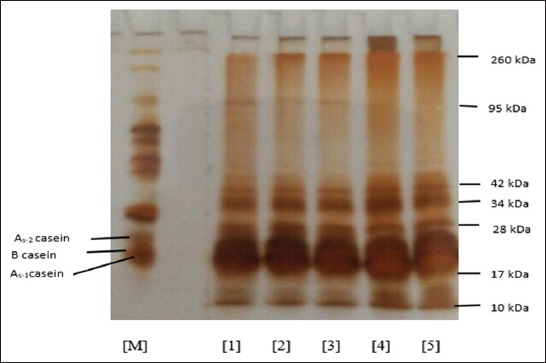
Proteolysis profile with sodium dodecyl sulfate-polyacrylamide gel electrophoresis of the cheese stored at frozen temperature for 60 days. Note: [M] = Marker; [1] = 0-day cheese storage; [2] = 15-day cheese storage; [3] = 30-day cheese storage; [4] = 45-day cheese storage; and [5] = 60-day cheese storage.

The proteolysis profile (Figure-3) in frozen-stored cheese for 60 days shows intact bands until the end of storage. Casein protein (α_s1_-casein, α_s1_-casein, and β-casein) of frozen-stored cheese does not experience any proteolysis, which is possible because the freezing gives minor influence on the cheese composition, including the cheese casein; the freezing does not damage the cheese protein structure, and hence, the proteolysis process occurs only partially. In goat cheese, the proteolysis process begins with a change in the α_s_-casein rather than in β-casein [25] because, in frozen-stored goat cheese, no proteolysis occurs until the 60^th^ day of storage.

The partial proteolysis occurring in the casein in electrophoresis pattern shows protein breakdown with lower molecular weight migrating faster and possibly related to the further degradation of the cheese peptide. The qualitative evaluation of goat cheese stored in frozen/freezer temperature with electrophoresis profile indicates that the cheese sample has homogeneity in the proteolytic profile, and hence, there was no band difference between storages. The casein breakdowns detected until the 60^th^ day of storage are α_s1_-casein, α_s2_-casein, β-casein, and κ-casein. Cheese is a source of α-casein and β-casein, which is broken down from peptides with high, medium, and low molecular weights such as the case of amino acid when it is hydrolized [16]. In goat cheese, β-casein is the main casein group, and this fraction is important to form and solidify the cheese [26]. α_s2_-casein exists in mammal’s milk in high amount in goat milk [27]. During the cheese production and storage processes, the peptides will be released by the proteolytic enzyme to be certain casein fraction. The breakdown of protein into κ-casein becomes important since κ-casein exists in the outer part of casein micelle which plays some role in the cheese stability and texture [28].

In standard condition, the electrophoresis mobility, β-casein is the main component of the casein breakdown product in goat milk, where the estimated percentage of the breakdown of the total caseins, i.e., for α-casein and β-casein in goat, is 5.6 and 38, respectively [29]. Goat milk has higher α_s2_-casein and κ-casein concentrations than cow milk.

## Conclusion

The physicochemical characteristics of cold-stored cheese are better than the cheese stored at frozen temperature. However, frozen-stored cheese produces lower FFA and ADV than cold-stored cheese and lipolysis occurs only partially. Frozen-stored cheese can maintain the number of LAB and lower total yeast as the contaminant factor in the cheese production process. The proteolysis of frozen-stored cheese is detected from the presence of α_s1_-casein, α_s2_-casein, β-casein, and κ-casein in the casein breakdown for 60-day storage.

## Authors’ Contributions

TS designed the research. JS and KW collected and prepared the milk sample. The cheese was made by TS, and then, chemical analyses were performed by all of the authors. JS and KW analyzed and interpreted the result. TS drafted the manuscript. All authors read and approved the final manuscript.
